# Molecular Determinants of Neurocognitive Deficits in Glioma: Based on 2021 WHO Classification

**DOI:** 10.1007/s12031-023-02173-4

**Published:** 2024-02-05

**Authors:** Kun Zhang, Tianrui Yang, Yu Xia, Xiaopeng Guo, Wenlin Chen, Lijun Wang, Junlin Li, Jiaming Wu, Zhiyuan Xiao, Xin Zhang, Wenwen Jiang, Dongrui Xu, Siying Guo, Yaning Wang, Yixin Shi, Delin Liu, Yilin Li, Yuekun Wang, Hao Xing, Tingyu Liang, Pei Niu, Hai Wang, Qianshu Liu, Shanmu Jin, Tian Qu, Huanzhang Li, Yi Zhang, Wenbin Ma, Yu Wang

**Affiliations:** 1 Department of Neurosurgery, Peking Union Medical College Hospital, Chinese Academy of Medical Sciences and Peking Union Medical College, Beijing, 100730 China; 2 School of Medicine, Tsinghua University, Beijing, 100730 China

**Keywords:** Glioma, Glioblastoma, WHO CNS5, Neurocognition, Genetic alteration

## Abstract

Cognitive impairment is a common feature among patients with diffuse glioma. The objective of the study is to investigate the relationship between preoperative cognitive function and clinical as well as molecular factors, firstly based on the new 2021 World Health Organization’s updated classification of central nervous system tumors. A total of 110 diffuse glioma patients enrolled underwent preoperative cognitive assessments using the Mini-Mental State Examination and Montreal Cognitive Assessment. Clinical information was collected from medical records, and gene sequencing was performed to analyze the 18 most influenced genes. The differences in cognitive function between patients with and without glioblastoma were compared under both the 2016 and 2021 WHO classification of tumors of the central nervous system to assess their effect of differentiation on cognition. The study found that age, tumor location, and glioblastoma had significant differences in cognitive function. Several genetic alterations were significantly correlated with cognition. Especially, *IDH*, *CIC*, and *ATRX* are positively correlated with several cognitive domains, while most other genes are negatively correlated. For most focused genes, patients with a low number of genetic alterations tended to have better cognitive function. Our study suggested that, in addition to clinical characteristics such as age, histological type, and tumor location, molecular characteristics play a crucial role in cognitive function. Further research into the mechanisms by which tumors affect brain function is expected to enhance the quality of life for glioma patients. This study highlights the importance of considering both clinical and molecular factors in the management of glioma patients to improve cognitive outcomes.

**Supplementary Information:**

The online version contains supplementary material available at 10.1007/s12031-023-02173-4.

## Introduction

Glioma is the most common type of primary malignant central nervous system tumor, accounting for approximately 59.2% (Ostrom et al. 2019). Compared to other tumors, glioma has a unique impact on neurocognitive function and approximately 60% of patients suffer cognitive impairments before or after treatment, including decreased attention, impaired memory, and decreased language skills (Cochereau et al. 2016; van Kessel et al. 2017), which severely impacts quality of life, especially for those with low-grade glioma who can often survive for more than 10 years. In addition, cognitive function may also reflect the subclinical lesion before imaging examination, which may help clinicians to detect insidious tumor progression earlier (Brown et al. 2006). At present, numerous scales have been used to assess cognitive function in patients with intracranial tumors, and as verified scales with high feasibility and validity, the Mini-Mental State Examination (MMSE, RRID:SCR_003681) (Folstein et al. 1975) and the Montreal Cognitive Assessment (MoCA) (Nasreddine et al. 2005) are the two main scales in cognitive function screening (Olson et al. 2008; Renovanz et al. 2018; Jia et al. 2021; Schiavolin et al. 2022).

It’s worth noting that the 2021 WHO classification of central nervous system tumors (WHO CNS5) now considers specific molecular alterations as crucial factors for glioma classification. This has been proven to better predict the prognosis of glioma patients and optimize individualized treatment strategies (Mortensen et al. 2022). However, there is currently a lack of relevant research on the cognitive status of patients with different types of gliomas under the new classification criteria.

Among the factors associated with cognitive impairment, several studies have suggested that cognitive decline may be due to occupational compression of brain functional areas, peritumoral edema, and the development of intracranial hypertension (Klein 2012). However, these explanations were powerful but not complete because they did not clarify the underlying internal mechanism. Few studies have shown that *IDH* mutation was associated with impaired cognitive function in glioma (Derks et al. 2019; Bunevicius et al. 2020; Pirozzi and Yan 2021). Also, it is suggested that the expression level of tumor-associated genes, such as *IDH-1*, *ATRX*, and *NLGN3*, was correlated with several cognitive domains (van Kessel et al. 2022). Nevertheless, numerous other molecules important to glioma necessitate further examination to elucidate their associations with cognition.

On the limitation of current research, the objective of the current study is to explore the factors affecting the patients’ preoperative cognitive function not only from the clinical perspective but also from the perspective of genetic alterations and tumor characteristics, such as metabolism and growth patterns, and for the first time under the new WHO CNS5 classification criteria. Also, we examined the ability of the WHO CNS4 and CNS5 to distinguish patients’ cognitive function.

## Method

### Study Participants and Eligibility Criteria

A non-randomized, prospective, longitudinal study was conducted. The aim of the study was to assess cognitive function in adult patients with diffuse glioma prior to glioma resection or biopsy surgery. Baseline information was obtained from medical record review at admission, including demographic information, tumor characteristics, radiographic data, and other significant medical and surgical history.

Eligibility criteria were age > 18 years, histologically diagnosed adult diffuse glioma according to WHO CNS5 classification criteria, and receiving surgery at Peking Union Medical College Hospital. Patients in agonal or deep coma stage with cognitive impairment and those who were unconscious during the evaluation process were excluded from this study. Perinatal women were also excluded.

### Assessment Questionnaires and Data Collection

The cognitive questionnaires applied were the MMSE (Folstein et al. 1975) and MoCA (Nasreddine et al. 2005), which were the most widely used cognitive ability scales in brain tumor patients. Higher scores on both the MMSE and MoCA are associated with better cognitive status, while years of education are associated with the assessment of cognitive impairment and dementia. The results of the cognitive questionnaires were entered individually into an online database, and each questionnaire contained a subset of independent items.

Clinical information was collected retrospectively from patients’ medical records and examinations. Clinical information included in the analysis included patient sex, age at diagnosis, body mass index (BMI), years of education, oncological history, tumor location, recurrent or not, and preoperative Karnofsky Performance Status (KPS) score. Recurrent is defined by Response Assessment in Neuro-Oncology (RANO) criteria (Wen et al. 2010; van den Bent et al. 2011).

### Tumor Pathology Data Collection

Histopathological and molecular pathological data were collected. Histopathological data were obtained from the report of the Department of Pathology of Peking Union Medical College Hospital, mainly including the Ki-67 index and WHO histological grade. Fifty-two molecular markers including *TERT*, *EGFR*, *CDK4*, *CDK6*, *CDKN2A*, *CDKN2B*, *MYB*, *FGFR2*, *FGFR3*, *PDGFRA*, *KRAS*, *BRAF*, *MET*, *MYBL1*, *IDH*, *MYCN*, *CIC*, and *ATRX* were screened in this study using the next-generation sequencing, polymerase chain reaction-based assay, and fluorescence in situ hybridization methods. These markers were selected by summarizing recently published studies on the prospects of differentiating glioma subtypes according to the updated WHO CNS5 classification, the mechanism of glioma development and prognostic factors. The genes included in the final correlation analyses were those with an alteration frequency of 10 to 90% in the tumor samples and correlated with at least one grading index. Gene alterations included mutation, deletion, amplification, amplification/deletion, and mix, according to Talevich et al. (2016). (See details in supplementary document 1.) The complete list of molecular markers is shown in Supplementary Table 1.

### Statistical Analysis

Baseline characteristics were expressed as frequency (*n*) for categorical variables and means ± standard deviations (SDs) for normally distributed continuous variables or medians plus interquartile range for non-normal distribution continuous variables. All continuous variables underwent a normality test.

Comparisons of categorical variables were performed using the chi-squared test. Student’s *t*-test was used to assess the differences between normally distributed continuous variables, while a non-parametric test was used for non-normal distribution continuous variables. Since the scores of MOCA and MMSE were not normally distributed, the non-parameter test was used to determine the difference of scores between patients with distinct clinical characteristics. Then multiple linear regression was used to determine the independent factor of cognition scores. At the same time, we displayed the disparities in scores by non-parameter test between the patients with or without glioblastoma, both under two classification criteria—WHO CNS4 and 5—which give new opportunities to evaluate their efficiency. Point-biserial was used to determine the correlation between genetic alterations and cognitive function scores, and the results were shown as both tables and heatmaps. The waterfall plot was used to illustrate genetic alterations in patients with different cognitive abilities. For most parameters, all patients were included in the analysis. However, only patients with complete data were included in the analysis for some variables. Statistical significance was considered when *p* < 0.05. Statistical analyses were performed using IBM SPSS Statistics 27.0. for Windows (SPSS Inc., Chicago, IL, USA) and RStudio (v1.1.463).

## Result

### Patient Characteristics

One hundred ten patients with adult diffuse glioma were enrolled from May 2018 to August 2022. All patients completed MoCA and MMSE assessment before surgery. There were 66 men and 44 women, with a mean age of 49.26 years old (range 19–79). The majority of included patients had received 6–12 years of education (*n* = 57, 51.8%). The KPS score of majority patients was ≥ 80 (*n* = 97, 88.2%), while the mean KPS score was 97.73. Considering the distribution of the tumor, there were 44 solitary lesions in frontal lobe and 28 in non-frontal lobe, while 38 tumors involved more than one lobe. For histopathology, there were 43 oligodendrocytomas, 17 astrocytomas, and 50 glioblastomas (GBMs). As the WHO CNS 5 classification criteria included more molecular characterization in defining GBM than the WHO CNS 4 criteria, we divided the enrolled patients into GBM and non-GBM groups according to both classification criteria, and demographic characteristics were reported separately. As the participating patients were different in each pathology classification category, we put forward the baseline information for each pathology category in Table 1.

**Table 1:** Baseline characteristics of all eligible patients

**Demographic characteristics**		**Total patients** **(*n* = 110)**	**WHO5 non-GBM** **(*n* = 49)**	**WHO5 GBM** **(*n* = 54)**	**WHO4 non-GBM** **(*n* = 57)**	**WHO4 GBM** **(*n* = 53)**
**Sex**	**Male**	66	29	31	36	30
	**Female**	44	20	23	21	23
**Age**	**<30**	11	6	5	8	3
	**30 ≤age<50**	43	28	11	31	12
	**50≤age<65**	37	14	21	15	22
	**≥65**	19	1	17	3	16
	**Average ± SD**	49.26 ± 14.81	43.12 ± 10.76	55.13 ± 16.05	43.35 ± 11.84	55.62 ± 15.15
**BMI**	**<18.5**	6	3	3	3	3
	**18.5≤BMI<24**	43	14	26	19	24
	**24≤BMI<28**	44	21	20	25	19
	**≥28**	16	11	4	10	6
	**Average±SD**	24.29 ± 3.66	25.17 ± 3.93	23.43 ± 3.34	24.85 ± 3.82	23.68 ± 3.42
**Years of education**	**<6**	15	5	6	7	8
	**6~12**	57	28	28	32	25
	**>12**	31	14	15	16	15
	**Unknown**	7	2	5	2	5
**KPS**	**<50**	4	0	4	0	4
	**50≤KPS<80**	9	1	7	2	7
	**≥80**	97	48	43	55	42
	**Average±SD**	97.73 ± 14.46	98.06 ± 5.38	88.79 ± 18.12	96.92 ± 7.54	88.21 ± 18.35
**Disease stage**	**Newly diagnosis**	91	42	43	48	43
	**Recurrence**	19	7	11	9	10
**Side of the tumor**	**Left hemisphere**	54	20	32	24	30
	**Right hemisphere**	52	25	22	29	23
	**Bilateral**	4	4	0	4	0
**Tumor location**	**Frontal lobe**	44	26	17	27	17
	**Temporal lobe**	12	4	7	5	7
	**Parietal lobe**	12	5	7	7	5
	**Occipital lobe**	4	1	3	1	3
	**Multiple lobes**	38	13	20	17	21
**Surgery option**	**Gross total resection**	81	37	41	39	42
	**Subtotal resection**	18	9	8	13	5
	**Biopsy**	11	3	5	5	6
**Histopathology**	**Oligodendrocytoma**	43	43	0	40	0
	**Astrocytoma**	17	6	0	17	0
	**Glioblastoma**	50	0	54	0	53
**WHO Grade**	**2**	-	28	0	35	0
	**3**	-	21	0	22	0
	**4**	-	7	54	0	53

*n* number, *GBM* glioblastoma, *BMI* body mass index, *KPS* Karnofsky Performance Status

### Clinical Factor Effect Cognitive Function

Significant differences in cognitive function were found between different age (MOCA *p* < 0.001, MMSE *p* < 0.001), sides (MOCA *p* = 0.004, MMSE *p* < 0.001), and patients with or without GBM (MOCA *p* = 0.035, MMSE *p* < 0.001), not only in total MOCA and MMSE scores, but also in most cognitive domains (Table 2). In contrast, there were no significant differences between gender, tumor recurrence, or frontal lobe involvement.

**Table 2:** Clinical factors effect cognitive function (non-parametric test)

		**Age**			**Sex**			**Side**			**Relapse**			**Glioblastoma**			**Front lobe involved**		
		≥ 65	< 65	*p*	*F*	*M*	*p*	Left	Right	*p*	Primary	Relapse	*p*	Yes	No	*p*	Yes	No	*p*
**MOCA**	**Total**	14 (10–22)	24 (21–27)	<0.001*	24 (20.5–27)	24 (17–26)	0.404	22 (12–25.5)	25 (22–27)	<0.001*	24 (17–27)	22 (16–25)	0.164	20 (14–25)	26 (13–28)	<0.001*	24 (18–26.5)	24 (16.5–27)	0.956
	**Visuospatial/** **Executive**	2 (1–4)	4 (3–5)	<0.001*	4 (3–5)	4 (2–5)	0.383	3 (1–4.5)	4 (3–5)	0.003*	4 (3–5)	3.5 (3–4)	0.457	3 (1–4)	4 (3–5)	<0.001*	4 (3–5)	4 (2–5)	0.376
	**Naming**	2 (2–3)	3 (3–3)	0.002*	3 (2–3)	3 (3–3)	0.439	3 (2–3)	3 (3–3)	0.166	3 (3–3)	3 (2–3)	0.188	3 (2–3)	3 (3–3)	0.018*	3 (2.5–3)	3 (2.5–3)	0.85
	**Attention**	4 (1–6)	6 (5–6)	0.001*	6 (5–6)	6 (5–6)	0.348	5 (2–6)	6 (5–6)	<0.001*	6 (5–6)	5 (5–6)	0.334	5 (3–6)	6 (5–6)	<0.001*	6 (5–6)	6 (4–6)	0.268
	**Language**	2 (1–2)	2 (2–3)	0.015*	2 (2–3)	2 (1–3)	0.81	2 (1–3)	2 (2–3)	0.006*	2 (2–3)	2 (1–2)	0.168	2 (1–2)	2 (2–3)	<0.001*	2 (2–3)	2 (1–3)	0.386
	**Abstraction**	1 (0–1)	2 (1–2)	0.007*	2 (1–2)	1 (0–2)	0.202	1 (0–2)	1 (1–2)	0.364	1 (0–2)	1 (0–2)	0.637	1 (0–2)	1 (0.5–2)	0.779	1 (1–2)	1 (0–2)	0.549
	**Memory**	0 (0–1)	3 (1–4)	0.002*	3 (0.5–4)	2 (0–4)	0.147	0 (0–3)	3 (2–4)	<0.001*	2 (0–4)	1 (0–3)	0.142	1 (0.5–3)	3 (2–4)	<0.001*	2 (0–4)	2 (0–4)	0.8
	**Orientation**	4 (2–6)	6 (5–6)	<0.001*	6 (5–6)	6 (5–6)	0.408	6 (4–6)	6 (6–6)	<0.001*	6 (5–6)	6 (5–6)	0.8	6 (4–6)	6 (5.5–6)	0.044*	6 (5–6)	6 (5–6)	0.847
**MMSE**	**Total**	21 (15–25.5)	28 (26–29)	<0.001*	28 (23–29)	27 (22.5–29)	0.408	25 (17.5–29)	28 (27–29)	0.004*	27.5 (23–29)	27 (21–29)	0.642	25.5 (20–29)	28 (20–29)	0.001*	27 (23–29)	27 (22–29)	0.641
	**Orientation**	8 (6.5–10)	10 (9–10)	0.001*	10 (9–10)	10 (9–10)	0.758	10 (7–10)	10 (10–10)	0.004*	10 (9–10)	10 (8–10)	0.387	10 (7–10)	10 (9–10)	0.035*	10 (9–10)	10 (9–10)	0.957
	**Working memory**	3 (1–3)	3 (3–3)	0.004*	3 (3–3)	3 (3–3)	0.714	3 (2–3)	3 (3–3)	0.005*	3 (3–3)	3 (2–3)	0.539	3 (2–3)	3 (3–3)	0.03*	3 (3–3)	2 (2–3)	0.127
	**Attention and Calculation**	2 (1–5)	5 (4–5)	0.003*	5 (3–5)	5 (3.5–5)	0.204	4 (1–5)	5 (4–5)	<0.001*	5 (3–5)	4 (2–5)	0.182	4 (1–5)	5 (4–5)	0.006*	5 (3–5)	5 (2.5–5)	0.569
	**Memory recall**	0 (0–1)	2 (1–3)	<0.001*	2 (1–3)	2 (0–3)	0.082	2 (0–3)	2 (1–3)	0.18	2 (0–3)	2 (0–3)	0.539	2 (0–3)	2 (1–3)	0.021*	2 (1–3)	2 (0–3)	0.525
	**Language**	7 (5.5–8)	9 (8–9)	<0.001*	9 (8–9)	8 (7–9)	0.067	8 (6–9)	9 (8–9)	<0.001*	9 (7–9)	9 (7–9)	0.96	8 (6–9)	9 (8–9)	<0.001*	9 (8–9)	9 (6.5–9)	0.154

*GBM* glioblastoma

*p*<0.05 as determined by *non-parametric test

Multiple linear regression analysis was then used to examine the effect of clinical factors on MMSE and MOCA scores. Age (*p* < 0.001), side (*p* = 0.007), and recurrence (*p* = 0.001) were significantly associated with MOCA. For MMSE, the influence of age (*p* < 0.001) and recurrence (*p* = 0.011) was statistically significant. Left cerebral hemisphere had a positive effect but did not reach significant (*p* = 0.055) (Table 3). For both MOCA and MMSE, age and relapse are independent factors.

**Table 3:** Clinical factors effect cognitive function (multiple linear regression)

	**MOCA**				**MMSE**			
**Variables**	***B***	***σ***	***β***	***p***	***B***	***σ***	***β***	***p***
**Interception**	40.598	5.091	——	<0.001	40.777	5.461	——	<0.001
**Age**	−0.253	0.044	−0.524	<0.001*	−0.178	0.047	−0.398	<0.001*
**Sex**	−0.344	1.094	−0.024	0.754	−0.656	1.185	−0.049	0.581
**Sides**	−3.071	1.112	−0.216	0.007*	−2.320	1.193	−0.177	0.055
**Relapse**	−4.983	1.524	−0.265	0.001*	−4.218	1.632	−0.244	0.011*
**GBM**	1.780	1.234	0.125	0.152	1.048	1.321	0.080	0.429
**Frontal lobe involved**	−1.134	1.149	−0.079	0.326	−1.517	1.237	−0.114	0.223

*GBM* glioblastoma, *B* non-standardized coefficient, *β* standardization coefficient, *σ* standard deviation

*p*<0.05 as determined by *multiple linear regression

### Comparison of WHO CNS4 and WHO CNS5 from Cognitive Aspect

Comparing the MOCA score under WHO CNS 4 and CNS 5, a significant difference was found in the “naming” domain between GBM and non-GBM groups defined by WHO CNS 5, but not tenable for WHO CNS 4. However, most cognitive domains, such as visuospatial/executive, attention, language, memory, orientation and total score, showed significant differences between GBM and non-GBM groups, regardless the classification criteria. As for MMSE, there were similar results for both classification criteria (Table 4).

**Table 4:** Comparison of WHO CNS4 and WHO CNS5

			**WHO CNS5**			**WHO CNS4**		
		**Total (*n* = 110)**	**GBM** **(*n* = 54)**	**Non-GBM** **(*n* = 56)**	***p***	**GBM** **(*n* = 50)**	**Non-GBM** **(*n* = 60)**	***p***
**M** **O** **C** **A**	**Visuospatial/Executive**	4 (3, 5)	3 (1, 4)	4 (3.25, 5)	<0.001*	3 (1, 4)	4 (3, 5)	<0.001*
	**Naming**	3 (2, 3)	3 (2, 3)	3 (3, 3)	0.018*	3 (2, 3)	3 (3, 3)	0.104
	**Attention**	6 (5, 6)	5 (3, 6)	6 (5, 6)	<0.001*	5 (2.75, 6)	6 (5, 6)	<0.001*
	**Language**	2 (1.75, 3)	2 (1, 2)	2 (2, 3)	<0.001*	2 (1, 2)	2 (2, 3)	0.002*
	**Abstraction**	1 (0, 2)	1 (0, 2)	1 (0.25, 2)	0.779	1 (0, 2)	1 (1, 2)	0.805
	**Memory**	2 (0, 4)	1 (0, 3)	3 (2, 4)	0.001*	1 (0, 3)	3 (1, 4)	0.002*
	**Orientation**	6 (5, 6)	6 (3.75, 6)	6 (5.25, 6)	0.044*	6 (3, 6)	6 (6, 6)	0.007*
	**Total**	25 (18, 27)	21.5 (15, 25.25)	27 (24, 28)	<0.001*	22 (12.75, 26)	26 (23, 28.75)	<0.001*
**M** **M** **S** **E**	**Orientation**	10 (9, 10)	10 (7, 10)	10 (9, 10)	0.051	10 (7, 10)	10 (9, 10)	0.008*
	**Working memory**	3 (3, 3)	3 (2, 3)	3 (3, 3)	0.045*	3 (2, 3)	3 (3, 3)	0.135
	**Attention and Calculation**	5 (3, 5)	4 (1, 5)	5 (4, 5)	0.009*	4 (1, 5)	5 (4, 5)	0.015*
	**Memory recall**	2 (0.5, 3)	2 (0, 3)	2 (1, 3)	0.029*	1 (0, 3)	2 (1, 3)	0.021*
	**Language**	9 (7, 9)	8 (6, 9)	9 (8, 9)	<0.001*	8 (6, 9)	9 (8, 9)	<0.001*
	**Total**	27 (23, 29)	26 (20, 29)	28 (27, 30)	0.002*	26 (19, 29)	28 (26.25, 30)	0.001*

*n* number, *GBM* glioblastoma

*p*<0.05 as determined by *non-parameter test

### Genetic Alterations Associated with Cognitive Function in Patients

**Fig. 1:**
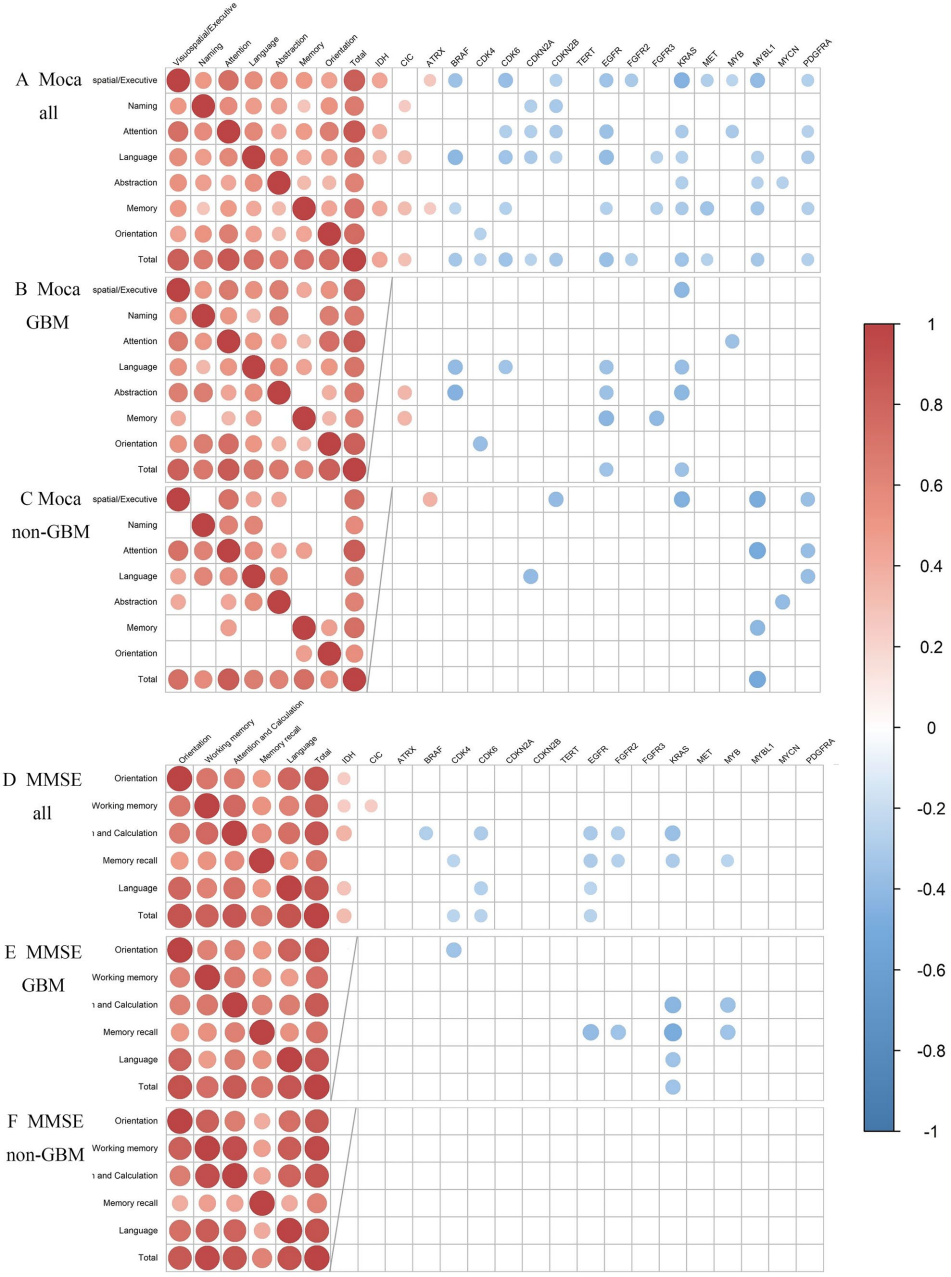
Correlation between genetic alteration and MOCA score and MMSE score. Red for positive correlations and blue for negative correlations, with dots representing significant correlations. The size and color depth of the dots are proportional to the correlation coefficient: **A**, **D** Result for all patients. **B**, **E** Result for patients with GBM. **C**, **F** Result for patients with non-GBM

We performed genetic sequencing on tumor samples from 67 patients. Significant correlations were found between many genetic alterations and MOCA total score also each cognitive domain. *IDH*, *CIC*, and *ATRX* are positively correlated with MOCA total scores also each domain in all patients, but negative most other genes are negatively correlated with MOCA score. Furthermore, although the number of genes associated with MOCA was reduced in GBM patients compared to all patients, *CIC* remained positively correlated with some cognitive domains, while *ATRX* lost significance. Negative correlations between *EGFR*, *KRAS*, and MOCA total scores were still maintained, and correlations with memory and abstraction were improved. In contrast, in non-GBM patients, *MYBL1* and *PDGFRA* were correlated significantly with improved abstraction (see Fig. 1A to C).

As for MMSE, there was also a significant correlation between genetic alterations and total scores as well as cognitive domains. In all patients, *IDH* alteration was positively correlated with MMSE score, and *CIC* was positively correlated with working memory score, which was consistent with the result for MOCA. In patient with GBM, negative correlations were found between *KRAS* alteration and many cognitive domain scores, which was also improved compared to the result for all patient. For *EGFR*, only the correlation with memory recall remained. And the correlation was also increased compared to the result for all patients, which is consistent with the result for MOCA. In contrast, no correlation was found between genetic alterations and MMSE scores in non-GBM patients. And regardless of the patient population, the number of genetic changes that correlated with MMSE was significantly reduced compared to MOCA. And the consistency within the scale itself was high across all cognitive domains (see Fig. 1D to F). Supplementary Tables 2 and 3 show details of correlation values.

**Fig. 2:**
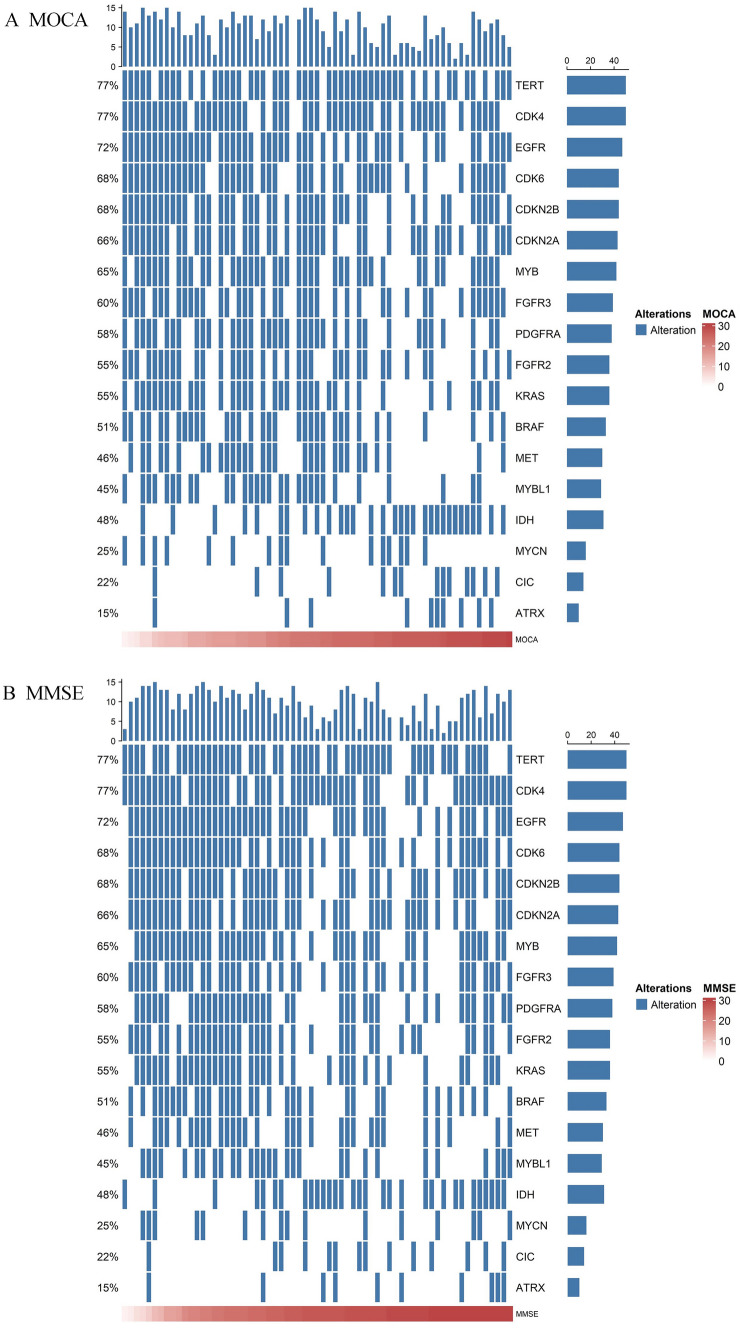
Waterfall plot of genetic alterations and MOCA scores and MMSE scores for all patients

To further show the correlation between genetic alterations and cognition, we plotted a waterfall of patients’ genetic alterations and cognitive scores. We found that among the 18 genes we screened, patients with a low number of genetic alterations tended to have higher MOCA scores, except for *IDH*, *MYCN*, *CIC*, and *ATRX*. The same pattern was shown in MMSE (see Fig. 2A and B). However, for the patient subgroup, the correlation between cognition and genetic alterations was significantly weaker, especially in non-GBM subgroup (Supplementary Fig. 1).

## Discussion

In this prospective study, we described several variables that influence patients’ cognitive function, from both clinical and molecular aspects, especially genetic alterations of glioma. In addition, we compared neurocognitive deficits between GBM and non-GBM patients using two classification criteria, WHO CNS 4 and CNS 5. We found that both criteria discriminated well between the two groups. Genetic alterations were associated with several cognitive functions, and their association was stronger in GBM patients than in non-GBM patients. Although most gene alterations were negatively correlated with cognitive function, the correlation of *IDH*, *CIC*, and *ATRX* was positive.

Consistent with previous studies (Yoshii et al. 2008; Miotto et al. 2011; Noll et al. 2015), our results showed that cognitive impairment was more severe in patients with left-side tumor, GBM, and in elder patients. High-grade glioma patients have worse language ability, processing speed, and executive function (Noll et al. 2015), which may be due to greater tumor momentum, or rapid growth. For, slow-growing tumors allow patients more time to shift affected cognitive functions to unaffected brain regions. Besides, studies revealed that patients with high-grade and low-grade gliomas displayed varying responses to brain functional connectivity and neuroplasticity. And this response is found independent from the patient’ age, sex, tumor location, and volume (Yuan et al. 2020). Connectome studies have also demonstrated the global effect of tumors on the brain (Hart et al. 2019). At the same time, some studies suggested that tumor volume is not a major factor affecting patient cognition (Smits et al. 2015; Yuan et al. 2020). Regarding age, our study discovered that cognitive function declines with age in patients with glioma, which may be associated with the increased frequency of high-grade gliomas in the elderly population. Besides, research has shown that the whole-brain functional connectome changes dynamically with age (Cao et al. 2014). And, the molecular mutation profiles of tumors exhibit heterogeneity among patients of different ages (Zapotocky et al. 2018; Jean-Quartier et al. 2021). Together, these studies suggest that tumors have effects beyond their localized area, including long-distance and global effects. And the global effects of distinctive biological traits of tumors on patients of various ages also varies. Subsequently, we aim to clarify the tumor’s potential impact on neurological function from a more fundamental molecular perspective.

Our researcher found that patients with *IDH* mutations had higher MMSE and MOCA scores, which was consistent with previous studies. It has been reported that patients with *IDH1*-wild type glioma were more likely to have impairments in verbal memory, language, visual construction, and manual dexterity more frequently (Wefel et al. 2016), which may be due to the rapid proliferation of tumor cells and lack of compensatory functional reconstruction of the brain rather than metabolic regulation of the tumor microenvironment (van Kessel et al. 2017). Emma van Kessel et al. found that IDH status is associated with psychomotor speed, memory performance, and executive functioning (van Kessel et al. 2022). Also, Zhe Zhang et al., summarizing data from a total of 104 patients with primary supratentorial diffuse lower-grade glioma (DLGG), similarly found severe cognitive decline in terms of neurocognitive function performance in patients with IDHwt (Zhang et al. 2020). To explain this phenomenon, previous studies pointed out that IDH mutations lead to the production of 2-hydroxyglutarate, which is associated with good cognition (Venkatesh et al. 2019). Other studies found that patients with IDH-wt gliomas have lower overall functional connectivity, which is an important factor leading to poorer cognitive abilities (Derks et al. 2019). Shelli R Kesler et al. further confirmed, compared to IDH-mutant tumors, IDH wild-type tumors have significantly lower brain network global efficiency and degree, including in the medial frontal, posterior parietal, and subcortical regions (Kesler et al. 2017). Our study found that *ATRX* mutations showed preferential memory performance on the MOCA test, which may also be due to *ATRX* mutations favoring a slower tumor growth rate(van Kessel et al. 2017). Recently, a study involving 793 adult patients with diffuse glioma, suggested a strong correlation between ATRX status and patients’ memory performance (van Kessel et al. 2022). As a chromatin-binding protein, ATRX mutation leads to the loss of function, but it is still unclear whether these cognitive changes originate from this kind of alteration. In addition, *ATRX* alterations were found to be frequently present in neurofibromatosis (type 1)-associated high-grade astrocytoma and were associated with in a variety of functional (impaired cognition, attention deficits and autism spectrum disorder) abnormalities (Nix et al. 2020). Moreover, *ATRX* mutation is associated with alpha thalassemia X-linked intellectual disability syndrome, often manifested as generalized cognitive impairment (Valenzuela et al. 2021). It is worth noting that some studies have proposed that IDH and ATRX alterations are more common in low-grade gliomas (van Kessel et al. 2022). Therefore, it is essential to consider whether the cognitive changes associated with these genetic alterations are primarily driven by different rate of tumor growth. *CIC* forms a transcriptional repressor complex with the protein ataxin 1 (*ATXN1*), involving in brain development and autoimmunity regulation, implicating in neurodegenerative diseases (Lu et al. 2017; Park et al. 2017). Therefore, when discussing the impact of the CIC protein on cognition, it is crucial to consider both the CIC-ATXN1 complex and the function of ATXN1. Several studies have indicated that alterations in CIC-ATXN1 in mammals can lead to motor symptoms, and blocking them can improve ataxia (Lee 2020). Additionally, Spinocerebellar ataxia type 1 (SCA1), an adult-onset neurodegenerative disorder characterized by motor incoordination and cognitive decline, has been found in a close relationship with the modification of the ATXN1 protein (Nitschke et al. 2021). CIC mutations can also lead to disruptions in folate synthesis, further resulting in poor muscle tone and coordination (Cao et al. 2021). Previous studies have suggested that *CIC* mutated neurodegenerative disease was associated with attention deficit, hyperactivity, impaired learning, and memory,which has been further verified in both mouse models and patients (Exome Aggregation Consortium et al. 2016; Lu et al. 2017). Our study found that altered *CIC* in glioma was associated with better cognitive status; this raises the question of whether mutations in CIC affect the function of the CIC-ATXN1 complex, thereby inhibiting its neurotoxic effects. *RAS/MAPK* signaling pathway was implicated in a group of developmental disorders with cognitive deficits of variable severity called Noonan syndrome spectrum disorders (NSSDs) (Schubbert et al. 2007; Cesarini et al. 2009). Mutations in *KRAS*, an important member of *RAS/MAPK* pathway, have been observed in up to 5% of patients with NSSDs and related to severe intellectual disability (Schubbert et al. 2006; Pierpont et al. 2010; Wingbermühle et al. 2022). Our analysis showed that *KRAS* mutation was a broad risk factor causing multiple dysfunctions in glioma patients. Diffuse gliomas with *MYB/MYBL1* rearrangement were mainly presented in children (Wefers et al. 2020), and most of the patients had epileptic seizures together with neurological symptoms such as movement disorders, behavioral or memory changes (Titulaer et al. 2013; Quiroz Tejada et al. 2021). In fact, studies have highlighted a potential role for the *MYB* in terms of regulating cell cycle progression, thus causing rapid tumor growth (Persson et al. 2009). *EGFR* amplification has been reported in up to 45% of patients with glioblastoma (The Cancer Genome Atlas Research Network 2008), and numerous studies have demonstrated the important role of the *EGFR/PI3K/AKT/mTOR* signaling pathway in glioblastoma progression (Lee et al. 2017, 2018; Dai et al. 2018). It was involved in development of brain neurons, including dopaminergic neurons in the midbrain (Futamura et al. 2003). Some studies have suggested that *EGFR* regulates the mechanism of cellular senescence via excessive activation of *RAS* and the *RAS-BRAF-ERK1/2* signaling axis (Shang et al. 2020), providing new insights into the regulatory mechanism of *EGFR*, but further studies are needed to clarify the underlying mechanism of its direct influence. In craniopharyngiomas (CPs), specifically the papillary subtype (PCP), BRAF gene mutations are often detected, potentially leading to cognitive impairments and attention deficits (Erfurth 2023). More direct evidence comes from Emily Schroeder et al.’s study (2016), which illustrated in patients with bipolar I disorder that reduced BRAF protein expression in olfactory neuroepithelial progenitor cells (ONPs) can induce apoptosis via the MEK/ERK signaling pathway. Previous studies have demonstrated that TERT may protect against Alzheimer’s disease (AD) by lowering levels of reactive oxygen species (ROS) and preventing oxidative harm (Kuan et al. 2023). Furthermore, it has been discovered that in mouse models, TERT maintenance leads to the enhancement of the gene network responsible for controlling synaptic signaling and learning processes, which are significant for the preservation of cognitive functions (Shim et al. 2021). Meanwhile, TERT methylation impacts social functioning in patients with panic disorder by regulating the function of the left postcentral gyrus (Ding et al. 2022). Both in low-grade and high-grade gliomas, TERT mutations are linked to the subgroup with the worst prognosis (Eckel-Passow et al. 2015), (Suzuki et al. 2015), which aligns with the tumor maintenance role of telomerase. However, TERT alterations did not show a correlation with cognition in our study. This could stem from the bidirectional effects of TERT mutations, where the neuroprotection and tumor growth effects counteract each other. Furthermore, the fibroblast growth factor receptor (FGFR) gene, frequently altered in cancer, particularly in diffuse gliomas (64.3%) (Dono et al. 2023), has been found played a potential role in neural architecture in central demyelinating diseases (Zhang et al. 2023). FGFR proved to help manage neuroinflammation and promote nerve repair and recovery of motor function in multiple sclerosis (MS) patients (Zhang et al. 2023). It can also promote the creation of new neurons in the hippocampus, thus enhancing cognition (Grońska-Pęski et al. 2021). However, activation of the FGFR promotes tumor proliferation in various signaling pathways (Babina and Turner 2017), potentially lead to cognitive decline in patients. In our study, there was no correlation between cognitive function and FGFR gene alterations, which might be attributed to the gene’s dual role in promoting neurological function and tumor growth. Additionally, our study solely investigated genetic alterations, excluding diverse mutation types that could influence signaling pathways differently. MYCN, a member of the MYC proto-oncogene family, is a transcription factor that controls the expression of a number of target genes, thereby regulating fundamental cellular processes, including proliferation, apoptosis, and differentiation (Westermark et al. 2011). Research on MYCN is primarily focused on neuroblastoma, and its proliferation is linked to undifferentiated morphology and a worse prognosis (Matthay et al. 2016). No studies have been conducted to demonstrate its particular function on cognitive ability in brain tumor patients. Similarly, CDKN, involved in cell cycle regulation and frequently altered in patients, lacks studies examining its impact on neurons or cognition.

The multifaceted roles of these genes highlight the complexity of understanding the factors contributions to cognitive function in the context of brain tumors. Our study highlighted the effect of genetic alterations on cognition, but many other aspects of cognition need further explored. On the one hand, as mentioned above, the size and location of the tumor and the occupying effect caused by edema also have an impact on the local brain function. On the other hand, there is still no standardized tool for cognitive assessment of glioma patients, and the impact of cognition dysfunction on quality of life needs to be assessed detailly. For patients with low-grade gliomas, whose survival is relatively long, protecting cognition is important in the long term, whereas for patients with high-grade gliomas, ensuring an acceptable quality of life is also critical. Based on the reaches so far, when considering the factors affecting patients’ cognition, we should take into account both macroscopic such as location and grade, and microscopic characteristics of the tumors.

Our strength was that the prospective research showed the impact of the tumor on cognition preoperatively. The study was based on the new WHO CNS 5 criteria and investigated the association between molecular characteristics and patients’ cognitive function from the genetic perspective, making a brand-new attempt to implement precision medical care in the future. However, there were still some limitations. First, we chose the MoCA and MMSE scales, which are already commonly used and have a high degree of reliability, but the scales currently used in the field of cognitive assessment in glioma were still not uniform and may lead to a decrease in the consistency of the findings. Second, we excluded patients who were unable to complete the assessment due to severe manifestation, which may increase selection bias.

The study discussed the relationship between molecular characteristics of glioma and cognitive function, bringing up a new aspect of what affects cognition in patients, and providing data support for the potential theoretical basis. Future studies may integrate multifactorial factors to identify individuals at high risk for cognitive impairment and provide them with cognitive rehabilitation training as early as possible to slow the process of cognitive impairment and improve the quality of patient survival. In addition, based on better understand about the relationship between these molecules and cognition, more targeted drugs on preserving patients’ cognition should also be developed rapidly. Also, determine whether peritumor region face the high possibility of potential cognitive plasticity can facilitate extended resection to benefit patients’ survival without functional cost. Furthermore, elucidating the molecular mechanisms that lead to cognitive decline in glioma patients can be a potential therapeutic breakthrough to improve cognitive reconstruction, which, in combination with new imaging techniques and clinical therapeutics, will lead to a better prognosis for patients.

### Supplementary Information

Below is the link to the electronic supplementary material.

Supplementary file1 (DOCX 412 KB)

## Data Availability

The datasets generated during and/or analyzed during the current study are available from the corresponding author on reasonable request.
