# Supplementary material for: Molecular Determinants of Neurocognitive Deficits in Glioma: Based on 2021 WHO Classification
Source: J Mol Neurosci. 2024 Feb 5;74(1):17. doi: 10.1007/s12031-023-02173-4 (PMC10844410; doi:10.1007/s12031-023-02173-4)
Supplement: Supplementary file 1 — Supplementary file1 (DOCX 412 KB) [file 12031_2023_2173_MOESM1_ESM.docx]

Supplementary Material

# Supplementary Figures and Tables

## Supplementary Figures

**
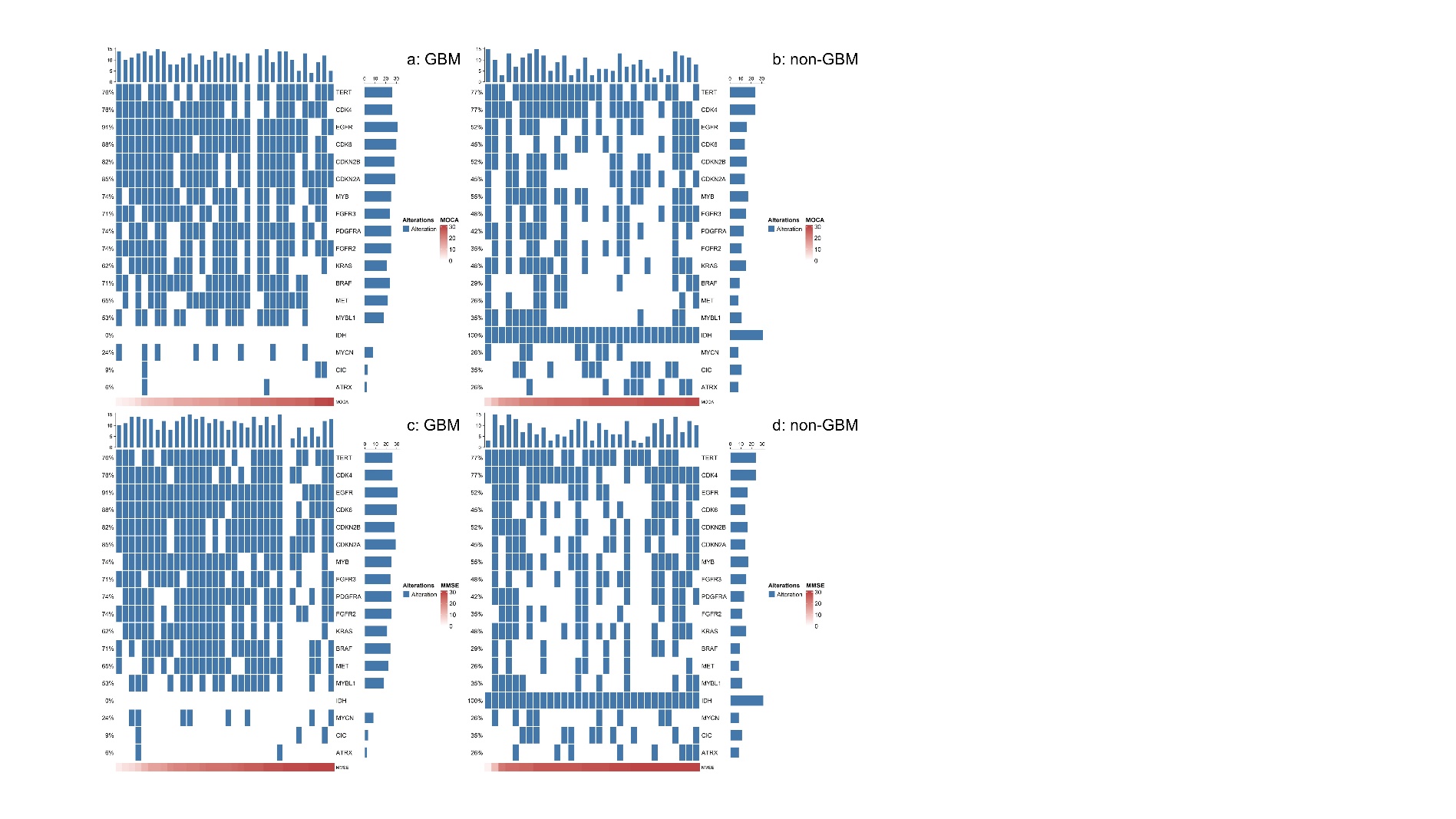
**

**Supplementary Figure 1.** Waterfall plot of genetic alteration. (A) shown as MOCA score for GBM patients; (B) shown as MOCA score for non-GBM patients; (C) shown as MMSE score for GBM patients; (D) shown as MMSE score for non-GBM patients

## Supplementary Tables

| **Number** | **Gene** | **Number** | **Gene** |
| --- | --- | --- | --- |
| 1 | ACVR1 | 27 | MYB |
| 2 | ATRX | 28 | MYBL1 |
| 3 | BCOR | 29 | MYC |
| 4 | BRAF | 30 | MYCN |
| 5 | CDK4 | 31 | NF1 |
| 6 | CDK6 | 32 | NOTCH1 |
| 7 | CDKN2A | 33 | NRAS |
| 8 | CDKN2B | 34 | NTRK2 |
| 9 | CIC | 35 | NTRK3 |
| 10 | EGFR | 36 | PDGFRA |
| 11 | FBXW7 | 37 | PEG3 |
| 12 | FGFR1 | 38 | PIK3CA |
| 13 | FGFR2 | 39 | PIK3CB |
| 14 | FGFR3 | 40 | PIK3R1 |
| 15 | FGFR4 | 41 | PPM1D |
| 16 | FUBP1 | 42 | PTEN |
| 17 | H3F3A | 43 | PTPN11 |
| 18 | HIST1H3B | 44 | RB1 |
| 19 | HIST1H3C | 45 | SMARCA4 |
| 20 | IDH1 | 46 | SMARCB1 |
| 21 | IDH2 | 47 | TERT |
| 22 | KIT | 48 | TOP3A |
| 23 | KMT5B | 49 | TP53 |
| 24 | KRAS | 50 | TSC1 |
| 25 | MAP2K1 | 51 | TSC2 |
| 26 | MET | 52 | YAP1 |

**Table S1 |** Gene panel of glioma

| **MOCA** | **Visuospatial/Executive** | | **Naming** | | **Attention** | | **Language** | | **Abstraction** | | **Memory** | | **Orientation** | | **Total** | |
| --- | --- | --- | --- | --- | --- | --- | --- | --- | --- | --- | --- | --- | --- | --- | --- | --- |
|  | **r** | **p** | **r** | **p** | **r** | **p** | **r** | **p** | **r** | **p** | **r** | **p** | **r** | **p** | **r** | **p** |
| **IDH** | .431** | <0.001 | 0.215 | 0.086 | .395** | 0.001 | .342** | 0.005 | 0.095 | 0.451 | .410** | 0.001 | 0.233 | 0.062 | .434** | <0.001 |
| **TERT** | -0.067 | 0.594 | -0.169 | 0.179 | 0.007 | 0.954 | 0.044 | 0.727 | -0.123 | 0.33 | -0.064 | 0.614 | -0.128 | 0.311 | -0.083 | 0.509 |
| **ATRX** | .262* | 0.035 | 0.192 | 0.125 | 0.135 | 0.282 | 0.077 | 0.541 | 0.112 | 0.373 | .257* | 0.039 | 0.108 | 0.39 | 0.227 | 0.07 |
| **BRAF** | -.351** | 0.004 | -0.189 | 0.132 | -0.207 | 0.099 | -.413** | 0.001 | -0.201 | 0.108 | -.247* | 0.047 | -0.135 | 0.283 | -.321** | 0.009 |
| **CDK4** | -0.157 | 0.212 | -0.169 | 0.179 | -0.241 | 0.053 | -0.161 | 0.201 | -0.166 | 0.187 | -0.182 | 0.147 | -.264* | 0.033 | -.261* | 0.036 |
| **CDK6** | -.380** | 0.002 | -0.175 | 0.163 | -.283* | 0.022 | -.343** | 0.005 | -0.131 | 0.296 | -.276* | 0.026 | -0.165 | 0.188 | -.343** | 0.005 |
| **CDKN2A** | -0.132 | 0.295 | -.285* | 0.021 | -.285* | 0.021 | -.304* | 0.014 | -0.05 | 0.691 | -0.118 | 0.348 | -0.222 | 0.075 | -.256* | 0.039 |
| **CDKN2B** | -.279* | 0.025 | -.316* | 0.01 | -.300* | 0.015 | -.269* | 0.03 | -0.131 | 0.296 | -0.187 | 0.136 | -0.227 | 0.069 | -.321** | 0.009 |
| **CIC** | 0.234 | 0.06 | .258* | 0.038 | 0.182 | 0.147 | .330** | 0.007 | 0.218 | 0.082 | .324** | 0.009 | 0.11 | 0.384 | .300* | 0.015 |
| **EGFR** | -.346** | 0.005 | -0.22 | 0.078 | -.351** | 0.004 | -.389** | 0.001 | -0.228 | 0.068 | -.276* | 0.026 | -0.136 | 0.281 | -.369** | 0.002 |
| **FGFR2** | -.308* | 0.013 | -0.179 | 0.153 | -0.241 | 0.053 | -0.235 | 0.059 | -0.174 | 0.165 | -0.205 | 0.102 | -0.122 | 0.333 | -.280* | 0.024 |
| **FGFR3** | -0.208 | 0.096 | 0.009 | 0.943 | -0.181 | 0.15 | -.267* | 0.031 | -0.037 | 0.769 | -.281* | 0.023 | -0.129 | 0.305 | -0.233 | 0.062 |
| **KRAS** | -.441** | <0.001 | -0.135 | 0.284 | -.306* | 0.013 | -.270* | 0.03 | -.284* | 0.022 | -.305* | 0.014 | -0.045 | 0.723 | -.349** | 0.004 |
| **MET** | -.284* | 0.022 | -0.112 | 0.374 | -0.158 | 0.21 | -0.213 | 0.089 | -0.157 | 0.212 | -.342** | 0.005 | -0.053 | 0.674 | -.261* | 0.035 |
| **MYB** | -.245* | 0.049 | -0.071 | 0.571 | -.304* | 0.014 | -0.231 | 0.064 | -0.085 | 0.502 | -0.173 | 0.168 | -0.078 | 0.538 | -0.243 | 0.051 |
| **MYBL1** | -.395** | 0.001 | -0.175 | 0.163 | -0.195 | 0.119 | -.285* | 0.021 | -.264* | 0.033 | -.330** | 0.007 | -0.052 | 0.683 | -.321** | 0.009 |
| **MYCN** | -0.048 | 0.703 | -0.222 | 0.075 | -0.169 | 0.177 | -0.121 | 0.338 | -.263* | 0.034 | -0.049 | 0.697 | -0.055 | 0.663 | -0.148 | 0.239 |
| **PDGFRA** | -.280* | 0.024 | -0.144 | 0.254 | -.266* | 0.032 | -.303* | 0.014 | -0.218 | 0.081 | -.283* | 0.022 | 0.029 | 0.818 | -.277* | 0.025 |

**Table S2 |** Correlation(r) and significance(p) of figure 1 A(MOCA)

| **MMSE** | **Orientation** | | **Working memory** | | **Attention and Calculation** | | **Memory recall** | | **Language** | | **Total** | |
| --- | --- | --- | --- | --- | --- | --- | --- | --- | --- | --- | --- | --- |
|  | **r** | **p** | **r** | **p** | **r** | **p** | **r** | **p** | **r** | **p** | **r** | **p** |
| **IDH** | .245* | 0.049 | .249* | 0.045 | .363** | 0.003 | 0.209 | 0.095 | .292* | 0.018 | .324** | 0.009 |
| **TERT** | -0.105 | 0.404 | -0.097 | 0.442 | -0.142 | 0.258 | -0.133 | 0.291 | -0.114 | 0.365 | -0.139 | 0.271 |
| **ATRX** | 0.082 | 0.516 | 0.139 | 0.269 | 0.164 | 0.191 | 0.058 | 0.647 | 0.132 | 0.295 | 0.134 | 0.289 |
| **BRAF** | -0.03 | 0.81 | -0.197 | 0.115 | -.287* | 0.021 | -0.168 | 0.181 | -0.19 | 0.13 | -0.191 | 0.128 |
| **CDK4** | -0.227 | 0.069 | -0.138 | 0.273 | -0.221 | 0.077 | -.250* | 0.045 | -0.188 | 0.135 | -.245* | 0.049 |
| **CDK6** | -0.13 | 0.302 | -0.196 | 0.118 | -.296* | 0.016 | -0.204 | 0.102 | -.276* | 0.026 | -.256* | 0.039 |
| **CDKN2A** | -0.2 | 0.11 | -0.066 | 0.599 | -0.16 | 0.203 | -0.16 | 0.204 | -0.214 | 0.086 | -0.203 | 0.104 |
| **CDKN2B** | -0.171 | 0.173 | -0.086 | 0.497 | -0.208 | 0.097 | -0.099 | 0.433 | -0.21 | 0.093 | -0.197 | 0.116 |
| **CIC** | 0.119 | 0.343 | .246* | 0.048 | 0.238 | 0.056 | 0.035 | 0.781 | 0.184 | 0.141 | 0.189 | 0.131 |
| **EGFR** | -0.096 | 0.446 | -0.225 | 0.072 | -.305* | 0.013 | -.293* | 0.018 | -.250* | 0.045 | -.259* | 0.037 |
| **FGFR2** | -0.136 | 0.278 | -0.079 | 0.534 | -.284* | 0.022 | -.266* | 0.032 | -0.218 | 0.081 | -0.235 | 0.059 |
| **FGFR3** | -0.115 | 0.361 | -0.168 | 0.181 | -0.166 | 0.187 | -0.191 | 0.127 | -0.233 | 0.061 | -0.2 | 0.11 |
| **KRAS** | -0.033 | 0.792 | -0.182 | 0.146 | -.368** | 0.003 | -.290* | 0.019 | -0.234 | 0.061 | -0.244 | 0.05 |
| **MET** | -0.002 | 0.988 | 0.026 | 0.834 | -0.175 | 0.163 | -0.072 | 0.567 | -0.163 | 0.194 | -0.1 | 0.426 |
| **MYB** | -0.067 | 0.595 | -0.191 | 0.127 | -0.218 | 0.082 | -.245* | 0.049 | -0.138 | 0.272 | -0.183 | 0.144 |
| **MYBL1** | 0.059 | 0.64 | 0.01 | 0.94 | -0.166 | 0.186 | -0.131 | 0.297 | -0.155 | 0.219 | -0.088 | 0.488 |
| **MYCN** | 0.003 | 0.983 | 0.035 | 0.782 | -0.103 | 0.414 | -0.117 | 0.353 | -0.15 | 0.234 | -0.083 | 0.51 |
| **PDGFRA** | 0.025 | 0.842 | -0.045 | 0.722 | -0.206 | 0.1 | -0.049 | 0.697 | -0.176 | 0.161 | -0.107 | 0.394 |

^*, p<0.05; **, p<0.01.^

**Table S3 |** Correlation(r) and significance(p) of figure 1 D(MMSE)

# Supplementary document 1 Methods for DNA sequencing

**Step1. DNA extraction and Library preparation**

A total of 65 formalin-fixed paraffin-embedded (FFPE) tumor tissue was obtained from Peking Union Medical College Hospital (PUMCH). DNA extraction was performed with AllPrep DNA/RNA FFPE Tissue Kit (QIAGEN 56404). Nucleotide concentrations were determined by Qubit 4.0 Fluorometer (Thermo Fisher Scientific) using the dsDNA HS Assay Kit (Invitrogen, Q32854). Nucleotide purity was determined by Nanodrop 2000 spectrophotometer (Thermo Fisher Scientific). NGS libraries were generated with the NEBNext Ultra II DNA Library Prep Kit for Illumina (New England Biolabs) and the The KAPA Target Enrichment Probes package (Roche) was used to perform enrichment of targeted regions. The concentrations of the sub and final libraries were determined using Qubit 4.0. The quality control results showed that the fragment size of the library was about 350 bp and the total amount of libraries was >500 ng.

**Step2. Next generation sequencing and analysis of NGS data**

Sequencing runs were either performed on the NovaSeq6000 with loading concentration of 240 pm. Sentieon was used to perform sequence comparison. Clean Reads is about 5G，mapping rate>99%, average sequencing depth >500X and target capture rate is 75%. Base Quality Score Recalibration (BQSR) was used to corrected base quality. Single nucleotide polymorphisms(SNP) also insertions and deletions (indels) were detected with TNscope. Copy number variations(CNVs) were identified with Cnvkit. The screening criteria for CNV is as follow:

[1] Deletion: indicates a deletion of the segment, which can be interpreted as a copy number decrease; judgment condition: the ratio of copy number <= 1.5 bin >= 0.3.

[2] Amplification: indicates that the region is amplified, which can be interpreted as copy number increase; judgment condition: the ratio of copy number >= 2.5 bin >= 0.3.

[3] Amplification/deletion: indicates that the region is amplifying and deletion; judgment condition: the ratio of copy number >= 2.5 bin >= 0.3 and the ratio of copy number <= 1.5 bin >= 0.3.

[4] Mix: indicates that a copy number change has occurred in the segment, but it is not sure whether it is an amplification or a deletion; judgment condition: (ratio of copy number <= 1.5 bin + ratio of copy number >= 2.5 bin) >= 0.3

The cut off of variant allele frequency (VAF) for hotspot mutation is 5% and 10% for non-hotspot mutation. The variation should be in exon region and not be synonymous mutation. Also, the variation should not be contained in the non-oncology population database of Genome Aggregation Database(GnomAD), East Asian Healthy People Database, and the Southern Chinese Han Population Database.

# Supplementary document 2 Details for cognitive assessment

All of the patients involved were assessed during 2018-2022. All researchers contributed to the assessment work, but the majority of cognitive assessments were performed by several nurses in the neurosurgery department, namely Zhiyuan Xiao, Lijun Wang, Wenwen Jiang, and Dongrui Xu from 2018-2022. Additionally, Jiaming Wu, Xin Zhang, and Junlin Li, who were in their postgraduate studies, also participated in the assessment work from 2020-2022. Each researcher who participated in the assessment process received training on administering the MoCA and MMSE scales as well as completed a traineeship and internship before conducting the independent assessments. Each patient underwent evaluation from admission to the hospital until surgery, within 7 days prior to the surgery.

**4. Supplementary document 3** **Additional notes on education level in MoCA scale**

Initially, the MoCA scale was validated in a sample of individuals with approximately 13 years of formal education (Nasreddine et al. 2005). However, as the scale became more widely used, researchers discovered that several subtests of MoCA included tasks that could be influenced by education levels, such as clock drawing, cube copy, and calculation. Therefore, bias may be introduced when individuals with lower levels of education participate in the test. Consequently, accurate and carefully designed screening measures are needed to support the detection of cognitive impairment in illiterate and low-educated populations. Indeed, many studies have made efforts in this regard. Preliminary analyses indicate that individuals with 12 years or less of education tend to perform poorly on the MoCA, to correct for the educational impact, participants with 12 years or less of education receive an additional point on their MoCA total score (if < 30 points) (Nasreddine et al. 2005). Ziad SN and colleagues, by comparing MoCA test data from different groups, including one with education levels not exceeding 12 years, found that after adjusting for education, each group obtained equivalent scores on the MoCA(Nasreddine et al. 2005) . Additionally, Parunyou J et al. conducted cognitive assessments for a group with less than 5 years of education (N=85), further confirming that after adjusting for education, the MoCA demonstrates excellent validity and accurately screens for cognitive impairment.

Reference

Nasreddine ZS, Phillips NA, BÃ©dirian V, et al (2005) The Montreal Cognitive Assessment, MoCA: A Brief Screening Tool For Mild Cognitive Impairment: MOCA: A BRIEF SCREENING TOOL FOR MCI. J Am Geriatr Soc 53:695–699. https://doi.org/10.1111/j.1532-5415.2005.53221.x
